# Downstream particle counting quantifies exposure reduction by miniaturized electrostatic precipitation compared to fibrous filtration

**DOI:** 10.21203/rs.3.rs-10561619/v1

**Published:** 2026-08-13

**Authors:** Chinmaya Mutalik, Rachel Redmann, Sarah Bose, Julian Henley, Bryan Tassin, Amy Phou, Chad J. Roy

## Abstract

Conventional respirator testing emphasizes filtration efficiency, but this metric does not fully capture how many particles ultimately reach the wearer. In this study, a standard N95 respirator and three prototype miniaturized electrostatic precipitator (mEP) masks, Mask A, Mask B, and Mask C, were evaluated using controlled aerosols of 1 µm, 4 µm, and mixed 1 + 4 µm polystyrene latex (PSL) particles. Rather than relying only on efficiency percentages, we quantified absolute downstream particle concentrations to compare passive fiber-based filtration with externally powered electrostatic particle removal strategies. All respirators reduced downstream particle concentrations dramatically compared with the no-mask control, typically by two to three orders of magnitude. For 1 µm PSL (~6.7 × 10⁵ particles min-1 without protection), Mask C allowed only ~8.8 × 10² particles min-1 , compared with ~1.67 × 10³ particles min-1 for the N95. The N95 served as the benchmark reference across aerosol conditions. SEM imaging confirmed dense PSL deposition under no-mask conditions and markedly reduced downstream deposition after respirator filtration, while EDS spectra verified PSL composition. These findings show that reporting absolute particle counts provides a direct, exposure-relevant framework for evaluating respirator performance.

### 1. Introduction

Personal protective equipment, particularly personal respirators, are widely used to reduce inhalation of airborne particles and to limit the release of particles generated during exhalation. Most respirators achieve this protection through passive capture within fibrous filter substrates, which remain the predominant particle-collection mechanism in current respiratory protection. In this study, we compare this established filtration approach with an experimental miniaturized electrostatic precipitation system designed for integration into a personal respirator. Respirator efficiency ratings like "N95" are often summarized as collection efficiencies at a particular particle size cutoff (e.g., 95% efficiency for particles >0.3 μm for N95 respirators), but these percentages do not directly convey the number of particles that reach the wearer. These particular efficiencies may not be adequate in an environment filled with millions of aerosolized particles $min^{-1}$, even the remaining percentage not collected could translate to an enormous number of particles entering the breathing zone. This concept is critical in understanding real exposure, as shown in earlier comparative efficiency studies which reveal that laboratory percentages do not always reflect true protective outcomes [1-3]. A particle count-based approach provides a more direct and intuitive perspective. Instead of relying on percentage filtration, it measures the actual number of particles penetrating a respirator under defined conditions[4, 5]. This metric reflects the inhalation dose, i.e., the quantity that ultimately reaches the respiratory tract and therefore correlates more meaningfully with potential health effects. Recent aerosol filtration studies highlight how particle concentration, size distribution, humidity, and airflow resistance together shape the user's effective protection[6-10].

The importance of this approach grew during and after the COVID-19 pandemic, when the need for low-resistance but highly protective devices inspired the development of miniaturized electrostatic precipitator (mEP) respirators. Studies have shown that well-designed mEP systems can maintain comparable or superior removal efficiency while improving comfort and wear time[1, 11-14]. Unlike fiber-based N95s, these protypes mEP devices use an externally powered electrostatic field to charge particles and collect them on plates, potentially enabling strong particle removal at lower breathing resistance[15-19]. This distinction between passive mechanical filtration and externally powered electrostatic particle removal enables comparison of two fundamentally different respiratory protection strategies using exposure-relevant particle-count metrics. In this study, we built on this evolution by testing whether the experimental mEP would approximate the collection efficiency of a filter-based respirator by quantifying particle counts to demonstrate how a count-based evaluation bridges laboratory data and real-world exposure. We chose to size-specific polystyrene latex beads (PSLs) rather than bioaerosols to test whether either of the particle collection methods exhibited differential efficiencies based upon size. This study tests respirator performance in a more human-centered way of showing not just how effective a mask is in principle, but how much it truly protects in practice.

### 2. Methods

*Experimental Configuration*. **Figure 1** illustrates the experimental configuration and analytical workflow used to compare the passive N95 reference respirator with the externally powered miniaturized electrostatic precipitator (mEP) respirator prototypes (Mask A, Mask B, and Mask C).

Experiments were performed in a 16 L plexiglass aerosol chamber using monodisperse polystyrene latex (PSL) Dragon Green™ microspheres (nominal diameters of 1 μm and 4 μm; Bangs Laboratories, Inc., Fishers, IN, USA) were used as surrogate aerosol particles throughout the study. The PSL microspheres or beads were supplied as an aqueous suspension and aerosolized using a Collison nebulizer. Equal-volume mixtures of the 1 μm and 4 μm suspensions were prepared to generate mixed-particle aerosol challenges. We used Reynolds number analysis to determine the airflow conditions in the exposure chamber based on its shape and total flow rate (~16 L $min^{-1}$). The estimated Reynolds number (~100-150) indicated laminar airflow conditions within the exposure chamber, supporting stable aerosol transport during downstream particle-count measurements. The estimated Stokes numbers for both 1 μm and 4 μm PSL particles were calculated as <1, indicating that both particle sizes closely followed the airflow streamlines in the chamber, which supports the accuracy of the downstream particle-count measurements. The aerosols 1 μm, 4 μm, and a 1:1 mixture of both sizes represent a range of respirable particle diameters[20, 21]. The chamber's concentration stabilized for approximately three minutes before each test. A commercially available N95 filtering facepiece respirator (3M, St. Paul, MN, USA) served as the passive reference respirator. Three prototype miniaturized electrostatic precipitator (mEP) respirators (Masks A–C) equipped with external power supplies were evaluated and compared with a passive N95 respirator. The prototype miniaturized electrostatic precipitator (mEP) respirators are externally powered, face-mounted devices that remove aerosol particles by controlled electrostatic precipitation. Within each prototype, a corona-discharge emitter imparts electrical charge to particles entering through the respirator inlet, after which the charged particles are attracted to and deposited on an internal oppositely charged collector plate. Electronic voltage control is used to regulate the ionization process and maintain the intended collection performance under variations in environmental conditions. The underlying mEP design was previously characterized at an inlet airflow of 6.85 L $min^{-1}$ for particle-removal performance, electrical power requirements, and ozone generation. The present study compared three configurations of this externally powered mEP platform (Masks A–C) with a passive N95 reference respirator under identical controlled aerosol-exposure conditions.[1] Each condition (No mask, N95, Mask A, Mask B, and Mask C) was run five times under identical flow rates (~16 L $min^{-1}$ total flow, 2.83 L $min^{-1}$ sampling). A six-channel optical particle counter (TSI 9306-V2) recorded cumulative particle counts[9, 10, 22]. Measurements were recorded at a sampling flow rate of 2.83 L $min^{-1}$ within a total chamber airflow of approximately 16 L $min^{-1}$.Counts were normalized to particles $min^{-1}$ to estimate downstream exposure concentration. PSL morphology and composition were evaluated using SEM-EDS (Hitachi SU-3500).

To allow direct comparison between masks, raw counts were normalized to particle concentration (particles per minute) using:

$$\text{Particles min}^{-1} = \frac{\text{Total Particle Count over 5 Min}}{5}$$

Exposure Reduction Index (ERI) relative to the no-mask condition was calculated as:

$$\text{ERI} = \frac{\text{No Mask particles min}^{-1}}{\text{Mask particles min}^{-1}}$$

ERI uncertainty was calculated using standard propagation of error for a ratio:

$$\text{ERI} \pm \text{SD} = \text{ERI} \times \sqrt{\left(\frac{\text{SD}_{\text{No Mask}}}{\text{Mean}_{\text{No Mask}}}\right)^2 + \left(\frac{\text{SD}_{\text{Mask}}}{\text{Mean}_{\text{Mask}}}\right)^2}$$

For qualitative validation, scanning electron microscopy (SEM) was used to image collection surfaces after aerosol exposure, and energy-dispersive X-ray spectroscopy (EDS) verified PSL composition. SEM confirmed visible particle accumulation without masks and sparse deposition on downstream filters when any respirator was present. Statistical analyses and data visualization were carried out using GraphPad Prism.

### 3. Results

Across all aerosol challenges, the respirators produced substantial reductions in downstream particle load compared with the unprotected condition[23]. Mean downstream particle concentrations and Exposure Reduction Index (ERI) values across all respirator conditions are summarized in Table 1. **Figure 2** presents downstream particle concentrations for the 1 µm, 4 µm, and mixed 1 + 4 µm PSL aerosol challenges on a $\log_{10}$ scale. Across all aerosol types, Mask C showed the lowest downstream particle transmission among the three mEP prototypes. Its performance approached that of the N95 respirator, matching it closely in the 4 µm tests and showing lower downstream particle counts than N95 in the 1 µm condition. In the mixed-size aerosol challenge, the N95 showed the lowest downstream particle concentration overall, while Mask C showed the lowest value among the mEP prototypes.

**Figure 3** shows the Exposure Reduction Index (ERI) values for all aerosol challenges, with the no-mask condition as the baseline. The N95 respirator and the prototype miniaturized electrostatic precipitator (mEP) masks both produced large reductions in the concentration of particles downstream. Mask C had the most stable high ERI values across all particle sizes and electrostatic configurations. The N95 respirator, on the other hand, had the lowest downstream particle concentration overall in the mixed-size aerosol condition. These findings validate that ERI serves as an additional exposure-based metric for evaluating respirator performance beyond mere percentage filtration efficiency[7, 20, 21, 24].

Qualitative assessment of downstream particle deposition was performed using SEM, as shown in **Figure 4**. Dense PSL particle accumulation was observed on collection substrates under no-mask conditions, whereas markedly reduced downstream deposition was observed following filtration through both passive N95 and externally powered mEP respirators. These observations visually confirm the substantial reductions in downstream particle transmission measured using optical particle counting[20, 21, 25].

Representative SEM micrographs and corresponding EDS spectra of deposited PSL particles are shown in **Figure 5**. SEM imaging confirmed the expected spherical shape of both 1 μm and 4 μm PSL particles that were captured on methylcellulose substrates[26]. The EDS spectra that went with them showed elemental signatures that were mostly carbon, which is what you would expect from polystyrene latex. These observations corroborate the hypothesis that the collected downstream particles originated from the PSL aerosol challenge and affirm the efficacy of the methylcellulose thin-film substrate for deposition-based visualization and compositional verification[27, 28].

The average downstream particle concentrations and Exposure Reduction Index (ERI) values for all respirator conditions (**Table 1A**), all respirators reduced particle counts by several orders of magnitude in the 1 µm test; Mask C produced the lowest downstream particle concentration among the mEP prototypes (~$8.8 \times 10^2$ particles/min), closely followed by Mask A, whereas Mask B showed higher leakage. The N95 and Mask C exhibited the lowest downstream particle concentrations for 4 µm aerosols (**Table 1B**), while Masks A and B remained below the $10^3$ particles/min range. Inclusion of the N95 mixed-aerosol condition enabled direct cross-platform comparison, with the N95 showing the lowest downstream particle concentration overall and Mask C showing the lowest value among the mEP prototypes (**Table 1C**). Taken together, these results show that absolute particles count resolve meaningful performance differences between respirator configurations.

### 4. Discussion

A count-based approach redefines respirator evaluation by focusing on exposure rather than efficiency percentages. The conditions in the laminar-flow chamber and the low particle Stokes numbers further confirmed the stable transport of PSL aerosols during exposure testing[8]. In our study, both N95 and mEP masks reduced downstream particle counts by two to three orders of magnitude, demonstrating strong overall protection. However, the count data also resolved meaningful performance differences among devices, with Mask C showing the most consistent low downstream particle counts among the electrostatic prototypes. Such behavior aligns with earlier work showing that filtration varies with particle size and airflow conditions[2, 3, 22, 24]. Quantifying results as absolute particle counts gives a clearer understanding of exposure. Even a small efficiency difference of less than 1% can translate into thousands of additional inhaled particles in high aerosol environments. Expressing respirator performance as particles $min^{-1}$ bridges engineering test metrics with exposure interpretation[29-32]. The current comparison underscores the difference between passive fiber-based filtration in N95 respirators and externally powered electrostatic particle removal in mEP respirators[13, 16]. Although they operate through different mechanisms, both approaches reduced downstream particle concentrations by multiple orders of magnitude under controlled aerosol exposure conditions.

SEM and EDS data supported the count-based findings by showing dense PSL deposition on control surfaces and minimal particle accumulation downstream of respirators[20, 26]. Although PSL beads are inert, their aerodynamic similarity to bioaerosols makes them a reliable model for studying particle transport and filtration behavior[15, 33] . The particle-count framework also simplifies comparing fibrous and electrostatic technologies[13, 16]. Focusing on absolute particle transmission rather than marginal efficiency gains will help develop next-generation respirators that achieve both high protection and comfort to quantify respirator protection. Across various particle sizes, both N95 and mEP prototypes reduced aerosol load relative to unprotected conditions, while Mask C showed the most consistent performance among the electrostatic precipitation. Future respirator standards may benefit from incorporating count-based metrics alongside conventional efficiency to improve clarity, comparability, and public understanding of protective performance.

### 5. Conclusion

Absolute particle counts provide a straightforward and exposure-relevant way to quantify respirator protection. Across the tested particle sizes, both N95 and mEP prototypes reduced aerosol load by approximately two to three orders of magnitude compared with unprotected condition. Mask C showed the most consistent low downstream particle counts among the electrostatic prototypes and bench level performance relative to the N95 respirator across multiple aerosol conditions. Expressing respirator performance as particles $min^{-1}$ connects testing outcomes directly to human exposure risk. Future respirator standards should consider incorporating count-based criteria alongside conventional efficiency metrics to improve clarity, comparability, and public understanding of protective performance.

**Funding**

This study was supported by National Institiute of Health Grant No. R44AI174405 and also in part by the Tulane University Center for Airborne Infection and Transmission Science (TUCAiTS).

### Figure Legends

**Figure 1. Experimental configuration and analytical workflow for controlled PSL aerosol exposure and respirator performance evaluation.** Compressed air supplied a Collison nebulizer containing approximately 10 mL of PSL suspension to generate 1 μm, 4 μm, or mixed 1 + 4 μm aerosol populations. Aerosol-laden air was transported through metal tubing into a 16-L plexiglass exposure chamber containing either the passive N95 reference respirator (upper panel) or one of three externally powered prototype miniaturized electrostatic precipitator respirators (Masks A–C; lower panel). Downstream particle concentrations were measured using a six-channel optical particle counter (TSI 9306-V2) and summarized using the Exposure Reduction Index (ERI) relative to the no-mask condition. Aerosols collected on methylcellulose thin-film substrates were subsequently characterized by scanning electron microscopy (SEM) and energy-dispersive X-ray spectroscopy (EDS) to confirm particle deposition and carbon-dominant elemental signatures consistent with polystyrene latex.

**Figure 2.** Downstream particle concentrations ($\log_{10}$ scale) measured for (a, b) 1 μm PSL aerosols, (c, d) 4 μm PSL aerosols, and (e, f) mixed 1 + 4 μm PSL aerosols across respirator conditions (No mask, N95, Mask A, Mask B, Mask C). Panels (a, c, e) compare each respirator with the unprotected condition, whereas panels (b, d, f) compare respirator configurations relative to the N95 benchmark. Bars represent mean ± SD (n = 5). Statistical significance was assessed by one-way ANOVA followed by Tukey's multiple-comparisons test and is denoted as ns, $*p < 0.05$, $**p < 0.01$, $***p < 0.001$, and $****p < 0.0001$. All respirators reduced downstream particle transmission by multiple orders of magnitude relative to no mask, with Mask C showing the lowest downstream particle loads among the mEP prototypes in the 1 μm and mixed 1 + 4 μm aerosol challenges and benchmark-level performance relative to N95 across multiple particle-size conditions.

**Figure 3.** Exposure Reduction Index (ERI) values relative to the no-mask condition for (a) 1 μm PSL aerosols, (b) 4 μm PSL aerosols, and (c) mixed 1 + 4 μm PSL aerosols. ERI represents the fold reduction in downstream particle concentration relative to the no-mask condition. Bars indicate mean ERI values (n = 5), and error bars represent propagated uncertainty (ERI ± SD). The y-axis is displayed on a $\log_{10}$ scale. Across all particle sizes, both the N95 respirator and the prototype miniaturized electrostatic precipitator (mEP) masks substantially reduced downstream particle transmission, with Mask C showing the most consistent high reduction among the electrostatic configurations.

**Figure 4.** SEM micrographs showing dense PSL depositions on the no-mask control surface and markedly reduced downstream depositions on methylcellulose collection films following respirator filtration.

**Figure 5.** Representative SEM micrographs and corresponding EDS spectra of deposited PSL particles. Panels (a, b) show representative 1 μm and 4 μm PSL particle morphologies observed on substrate surfaces, and panels (c, d) show corresponding EDS spectra confirming carbon-dominant elemental signatures consistent with polystyrene latex.

### Table Legend

**Table 1A-C.** Mean downstream counts, particles per minute, Exposure Reduction Index (ERI), and propagated ERI uncertainty (ERI ± SD) relative to the no-mask condition (n = 5 per condition). The lowest downstream count for each aerosol condition is highlighted.

## Figure 1

Controlled PSL aerosol exposure and respirator performance validation workflow
N95 (Reference respirator: passive filtration)
1 Compressed air supply
2 Collison nebulizer (~10 mL fluid)
3 Aerosol transport (metal tubing)
4 16-L controlled exposure chamber (plexiglass)
5 Outlet tubing
6 Optical particle counter (TSI 9306-V2)
7 Exposure reduction (ERI) Fold reduction relative to no-mask control
8 Captured methylcellulose substrate
9 SEM image (PSL particles on substrate)
10 EDS spectrum (polystyrene composition; carbon-dominant signal)
PSL aerosols (1 µm, 4 µm, and mixed population)
N95
2 µm
Counts
Energy (keV)
Externally powered electrostatic precipitator respirators
Mask A
Mask B
Mask C
Aerosol flow
Airflow direction
Tubing connection
Metal tubing
Electrical connection (external power)
PSL: Polystyrene latex | ERI: Exposure Reduction Index | SEM: Scanning Electron Microscopy | EDS: Energy Dispersive X-ray Spectroscopy

## Figure 2

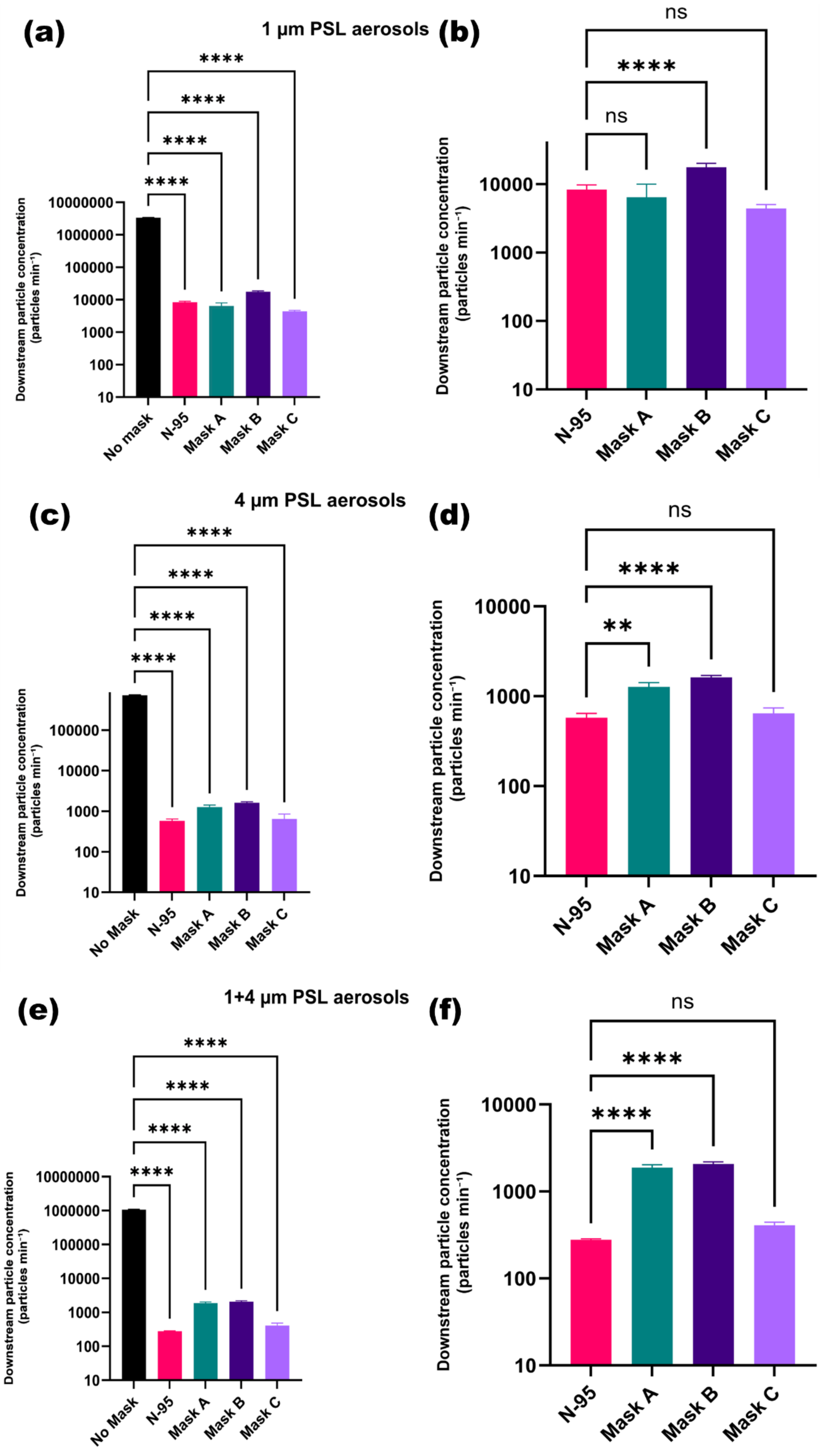

1 µm PSL aerosols
(a)
(b)
4 µm PSL aerosols
(c)
(d)
1+4 µm PSL aerosols
(e)
(f)
Downstream particle concentration (particles min⁻¹)
No mask
N-95
Mask A
Mask B
Mask C
ns
****
**

## Figure 3

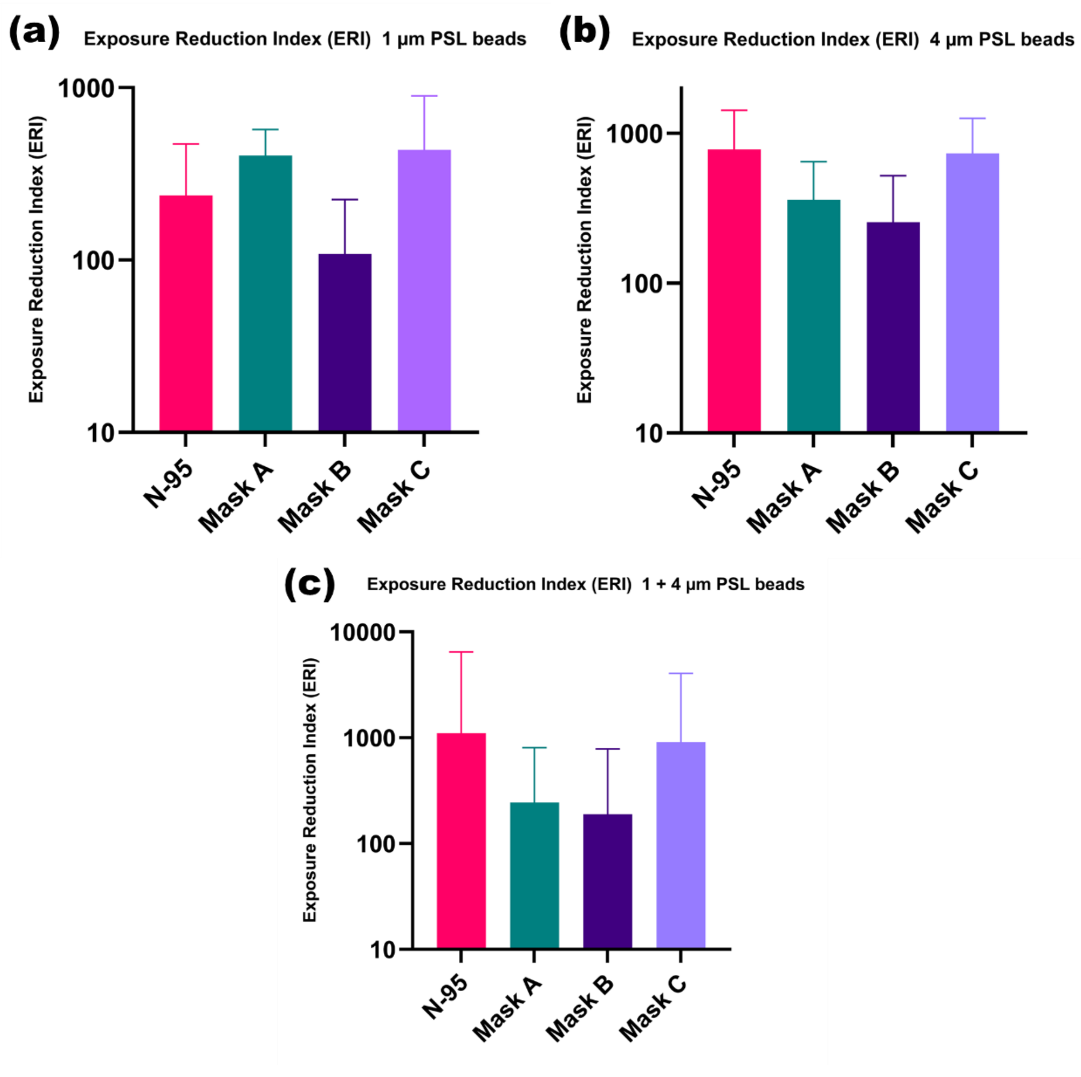

(a)
Exposure Reduction Index (ERI) 1 µm PSL beads
Exposure Reduction Index (ERI)
1000
100
10
N-95
Mask A
Mask B
Mask C
(b)
Exposure Reduction Index (ERI) 4 µm PSL beads
Exposure Reduction Index (ERI)
1000
100
10
N-95
Mask A
Mask B
Mask C
(c)
Exposure Reduction Index (ERI) 1 + 4 µm PSL beads
Exposure Reduction Index (ERI)
10000
1000
100
10
N-95
Mask A
Mask B
Mask C

Figure 4

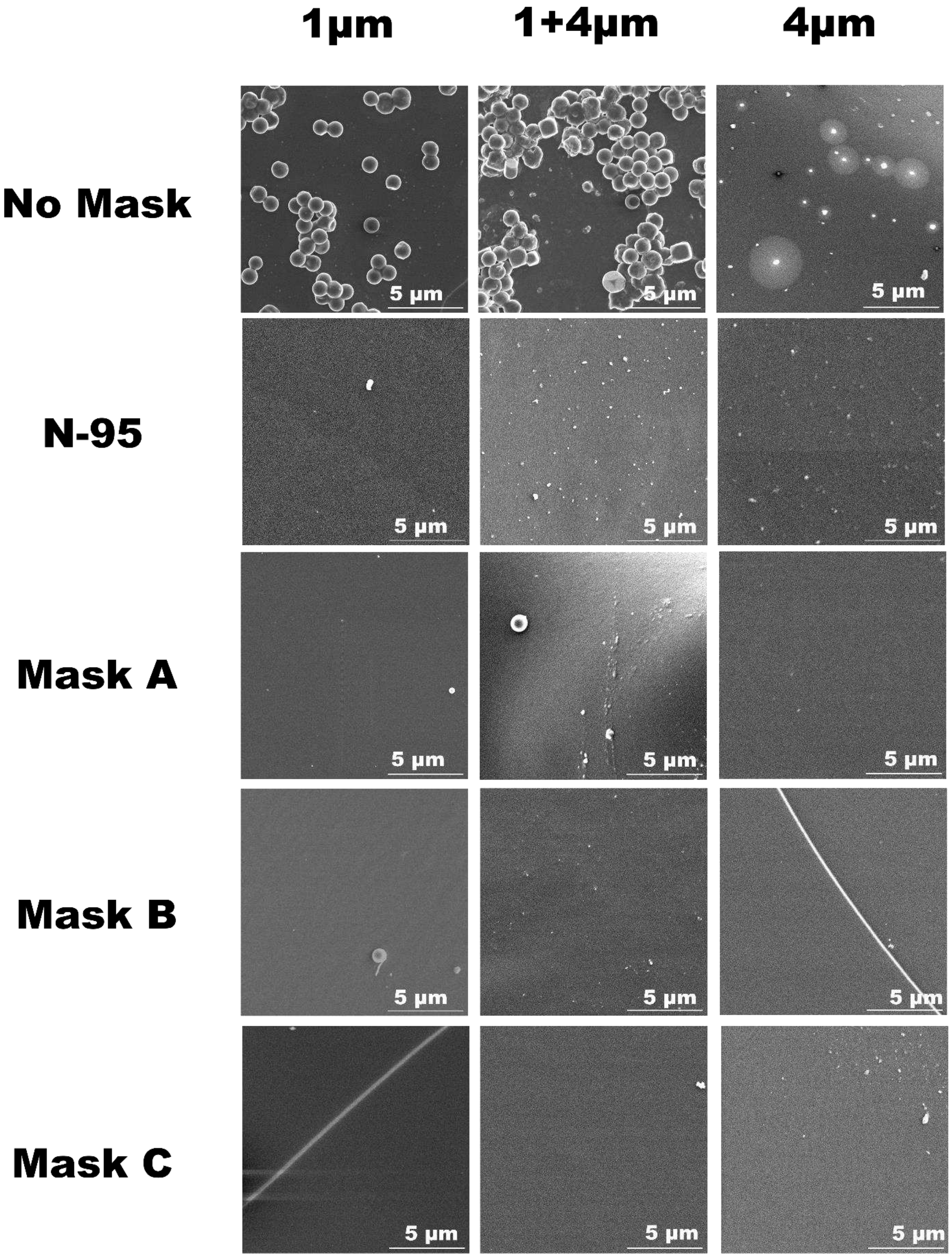

## Figure 5

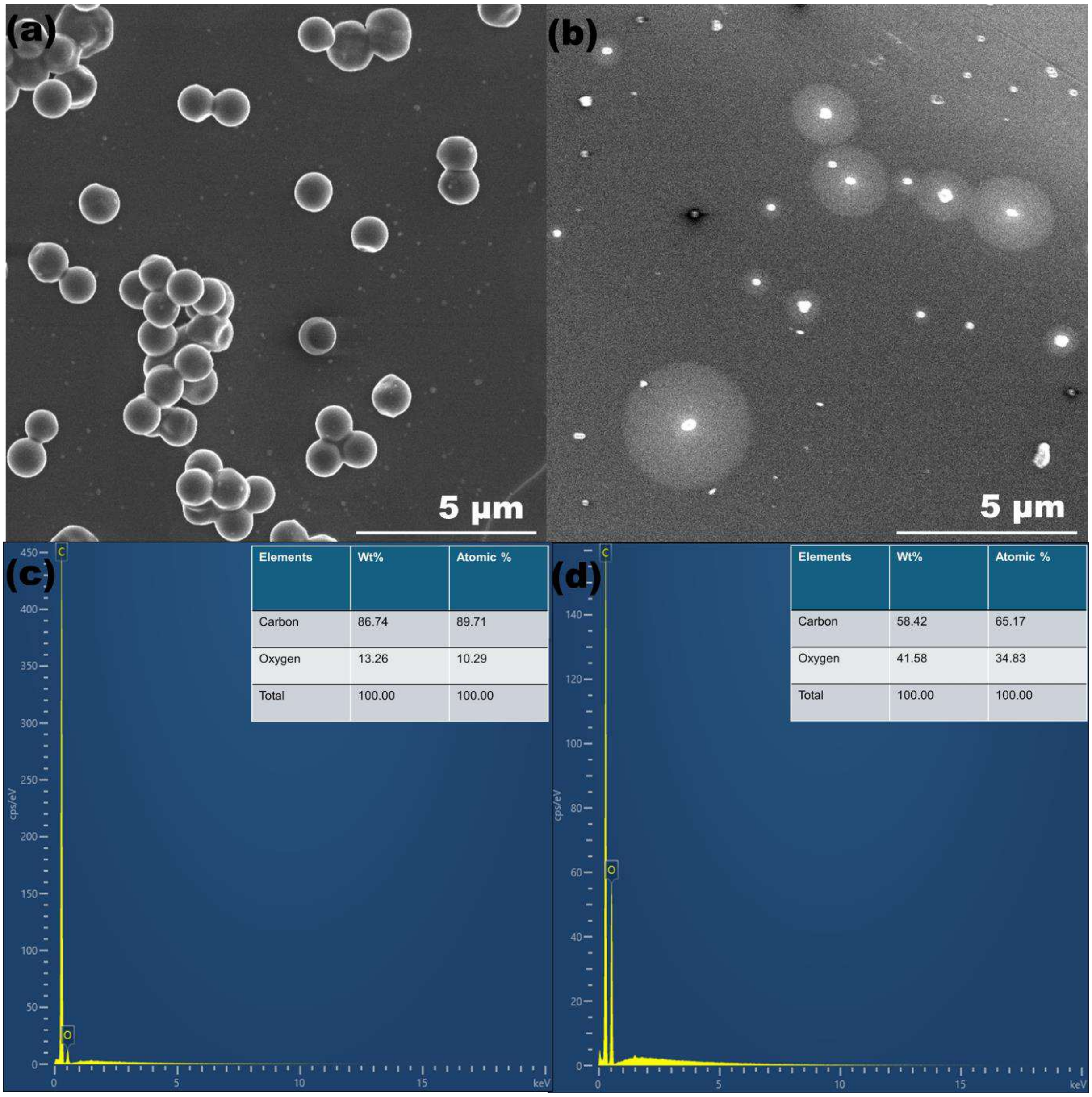

(a)
5 µm
(b)
5 µm
(c)
Elements Wt% Atomic %
Carbon 86.74 89.71
Oxygen 13.26 10.29
Total 100.00 100.00
C
O
cps/eV
450 400 350 300 250 200 150 100 50 0
0 5 10 15 keV
(d)
Elements Wt% Atomic %
Carbon 58.42 65.17
Oxygen 41.58 34.83
Total 100.00 100.00
C
O
cps/eV
140 120 100 80 60 40 20 0
0 5 10 15 keV

## Table 1A-C

#### A. 1 μm PSL Aerosols

| Mask | Mean Count (5 min) | SD | Particles min$^{-1}$ | ERI | ERI ± SD |
|---|---|---|---|---|---|
| Mask A | 6,824.8 | 3,490.8 | 1,365.0 | 492.0 | 267.8 |
| Mask B | 17,642.4 | 2,424.3 | 3,528.5 | 190.3 | 26.9 |
| **Mask C** | **4,406.4** | **619.8** | **881.3** | **762.1** | **109.9** |
| N95 | 8,317.8 | 1,413 | 1,663.6 | 403.7 | 69.8 |
| No Mask | 3,357,925.6 | 107,539.9 | 671,585.1 | 1 | - |

#### B. 4 μm PSL Aerosols

| Mask | Mean Count (5 min) | SD | Particles min$^{-1}$ | ERI | ERI ± SD |
|---|---|---|---|---|---|
| Mask A | 1,269.4 | 337.3 | 253.9 | 564.1 | 155.9 |
| Mask B | 1,612.8 | 209.7 | 322.6 | 444.0 | 66.8 |
| **Mask C** | **645.6** | **206.1** | **129.1** | **1,109.2** | **363.9** |
| **N95** | **577.4** | **144.6** | **115.5** | **1,239.9** | **324.5** |
| No Mask | 716,011.8 | 54,235.4 | 143,202.4 | 1 | - |

#### C. 1 + 4 μm PSL Aerosols

| Mask | Mean Count (5 min) | SD | Particles min$^{-1}$ | ERI | ERI ± SD |
|---|---|---|---|---|---|
| Mask A | 1,879.2 | 336.0 | 375.8 | 569.0 | 104.9 |
| Mask B | 2,069.6 | 265.4 | 413.9 | 516.6 | 70.2 |
| **Mask C** | **408.4** | **75.7** | **81.7** | **2,617.0** | **499.7** |
| **N95** | **277.6** | **19.2** | **55.5** | **3852.5** | **317.0** |
| No Mask | 1,069,059 | 48,588 | 213,811.8 | 1 | - |
